# Effects of Probiotic Culture Supernatant on Cariogenic Biofilm Formation and RANKL-Induced Osteoclastogenesis in RAW 264.7 Macrophages

**DOI:** 10.3390/molecules26030733

**Published:** 2021-01-31

**Authors:** Jae-In Jung, Seung-Min Baek, Trung Hau Nguyen, Jin Woo Kim, Chang-Ho Kang, Seonyoung Kim, Jee-Young Imm

**Affiliations:** 1 Department of Foods and Nutrition, Kookmin University, Seoul 02707, Korea; shinseo23@naver.com (J.-I.J.); doriya50@naver.com (S.-M.B.); 2 MEDIOGEN, Co., Ltd., Jecheon 27159, Korea; hau2807@naver.com (T.H.N.); valdicava@naver.com (J.W.K.); changho-kang@naver.com (C.-H.K.); clsrn3423@naver.com (S.K.)

**Keywords:** spent culture supernatant, *Streptococcus mutans*, biofilm formation, osteoclastogenesis

## Abstract

Postbiotics are a promising functional ingredient that can overcome the limitations of viability and storage stability that challenge the production of probiotics. To evaluate the effects of postbiotics on oral health, eight spent culture supernatants (SCSs) of probiotics were prepared, and the effects of SCSs on *Streptococcus mutans*-induced cariogenic biofilm formation and the receptor activator of the nuclear factor κB ligand (RANKL)-induced osteoclastogenesis were evaluated in RAW 264.7 macrophages. SCS of *Lactobacillus salivarius* MG4265 reduced *S. mutans*-induced biofilm formation by 73% and significantly inhibited tartrate-resistant acid phosphatase (TRAP) activity, which is a biomarker of mature osteoclasts in RAW 264.7 macrophages. The suppression of RANKL-induced activation of mitogen activated the protein kinases (c-Jun N-terminal kinase, extracellular signal-regulated kinase, and p38) and nuclear factor κB pathways, as well as the upregulation of heme oxygenase-1 expression. The suppression of RANK-L-induced activation of mitogen also inhibited the expression of transcriptional factors (c-fos and nuclear factor of activated T cells cytoplasmic 1) and, subsequently, osteoclastogenesis-related gene expression (tartrate-resistant acid phosphatase-positive (TRAP), cathepsin K, and matrix metalloproteinase-9).Therefore, SCS of *L. salivarius* MG4265 has great potential as a multifunctional oral health ingredient that inhibits biofilm formation and suppresses the alveolar bone loss that is associated with periodontitis.

## 1. Introduction

Probiotics are defined as viable microorganisms that exert health benefits on the host when they are consumed in sufficient amounts [1]. The beneficial health effects of probiotics are strain-specific and related to several fundamental mechanisms, such as competition with pathogens [2], reinforcement of the intestinal barrier function [3], modulation of immune responses [4], and production of neurotransmitters [5]. Postbiotics refer to diverse metabolites or microbial components produced by probiotics during fermentation. Postbiotics are an emerging functional ingredient since they effectively increase the efficacy of probiotics without causing problems related to colonization and viability [6,7]. Cell-free probiotic culture supernatants are characterized as a typical type of postbiotics. Considering that postbiotics are mixture of various compounds produced during fermentation, they display broad bioactivity spectrum and have synergistic activity between various compounds [8]. There has been an increase in the application of postbiotics in food processing, and the fabrication of postbiotic-incorporated edible antimicrobial film has been the subject of considerable interest [9,10].

Oral diseases, such as dental caries and periodontal diseases (gingivitis and periodontitis), are among the most prevalent global diseases and have physical, psychological, and economic consequences. Furthermore, normal oral function significantly affects the quality of life [11]. The oral cavity harbors more than 1000 microbial species and establishes a dynamic balance between microbial species [12]. Application of probiotics can contribute to the homeostasis of oral microflora by inhibiting the growth of periodontal pathogens and the modulation of the host immune responses [13].

*Streptococcus mutans* is a gram-positive facultative anaerobic bacterium that causes dental caries in the oral cavity. *S. mutans* produces dental plaque and creates acidic environment in the oral cavity [14]. Several studies have demonstrated that culturing *S. mutans* in the probiotic supernatants or coculturing *S. mutans* with probiotics reduced *S. mutans*-mediated biofilm formation [15,16,17]. Bacterial accumulation in the oral cavity also triggers inflammation in gingival tissue and increases inflammatory cytokines and oxidative stress, leading to periodontal diseases causing gum damage and alveolar bone loss [18,19]. Nuclear factor κB ligand (RANKL)-mediated osteoclast differentiation is also closely related to alveolar bone loss in periodontal disease [20]. Thus, suppression of osteoclast differentiation can ameliorate osteoclast-related disorders including periodontitis. Liu et al. [21] reported that the ethanol extract of *Lactobacillus paracasei* subsp. *paracasei* NTU 101-fermented skim milk significantly suppressed periodontal inflammation in lipopolysaccharide (LPS)-induced periodontitis rat model. Application (0.1 mL, 2 times/day for 14 days) of a *Weissella cibaria* suspension (5 × 10^9^ CFU/mL) on the gingival sulcus reduced alveolar bone loss in ligature-induced experimental periodontitis mice model [22].

The present study was conducted to identify potential biotherapeutic culture supernatants for the development of functional oral health ingredients. To achieve this goal, the effect of culture supernatants on the growth of *S. mutans* and biofilm formation were examined. Furthermore, we systemically investigated the effects of a selected culture supernatant (*Lactobacillus salivarius* MG4265) on RANKL-induced osteoclastogenesis and related molecular mechanisms.

## 2. Results and Discussion

### 2.1. Effect of Probiotic Strains on the Growth of S. mutans

**Figure 1:**
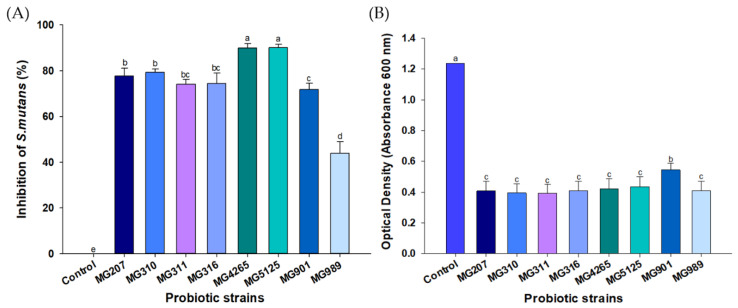
Effect of (**A**) probiotic strains and (**B**) their spent culture supernatants (SCSs) on the growth of *S. mutans*. Different letters (a–e) indicate significant difference at *p* < 0.05.

The potential inhibitory activity of eight probiotic strains on the growth of *S. mutans* was evaluated (Figure 1A). The tested probiotic strains significantly suppressed the growth of *S. mutans.* The highest inhibitory activity was obtained with *Lactococcus lactis* MG5125 and *L. salivarius* MG4265. When the spent culture supernatants (SCSs) of the probiotic strains were used, there was an overall significant reduction in the growth of *S. mutans*. SCS of *Lactobacillus fermentum* MG901 showed lower inhibitory effect on *S. mutans* compared to other SCSs, while no clear differences were observed among other SCSs (Figure 1B). Similarly, SCSs obtained from several *Lactobacillus* spp. with different metabolic patterns (*Lactobacillus salivarius*, *Lactobacillus casei, Lactobacillus plantarum,* and *Lactobacillus reuteri)* resulted in a significant reduction in the growth *S. mutans* as determined at 600 nm but no difference was detected among the samples [16]. These results suggest that the tested probiotic strains produced metabolites or extracellular components that have an ability to inhibit growth of *S. mutans*.

*S. mutans* is an acid-producing bacterium and can tolerate acidic environments. The glucosyltransferase-expressing ability to synthesize water-insoluble glucans contributes to its virulence traits [14]. *Lactobacillus* spp.-mediated growth inhibition of *S. mutans* was demonstrated in humans, and considerable differences were found in oral *lactobacilli* between individuals containing no dental caries and active caries [23].

Tong et al. [24] reported that *Lactococcus latis* exerted an antagonizing effect against growth of *S. mutans*, especially under nutrient-deficient conditions, and delayed the incidence of dental caries. Two *L. lactis* strains, HY 449 and ATCC 19435, significantly downregulated expression of the glucosyltransferase gene (*gtfs*) of S. mutans [25]. Two *L. salivarius* strains, K35 and K43, which have strong inhibitory activity on the growth and biofilm formation of *S. mutans*, have been identified [26]. The anticariogenic activity of these two strains is greater than that of *Lactobacillus rhamnosus* GG (LGG), and they effectively downregulate glucosyltransferase-encoding genes (*gtfB, gtfC*, and *gtfD*).

### 2.2. Effect of SCSs on S. mutans-Induced Preformed Biofilm Formation

**Figure 2:**
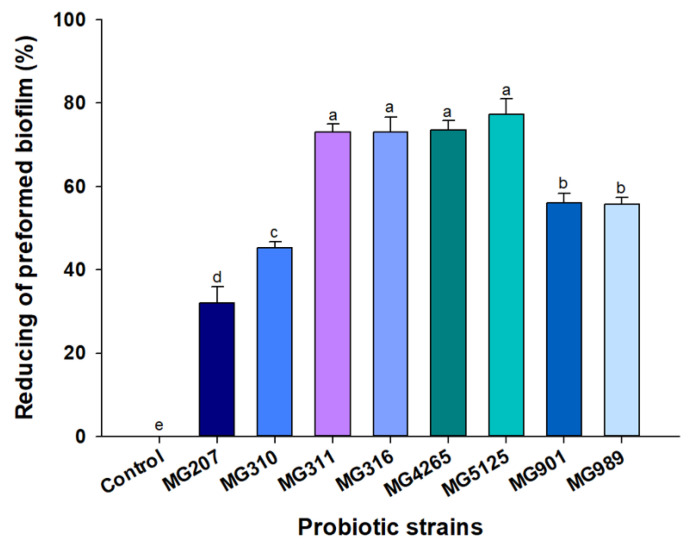
Reducing activity of SCSs of probiotic strains on *S. mutans*-induced biofilm. Different letters (a–e) indicate significant difference at *p* < 0.05.

Numerous studies have reported that *lactobacilli* produce various secondary metabolites that have been characterized as antibacterial substances, such as organic acids (primarily acetic acid and lactic acid), peptides (biosurfactant and bacteriocins), and hydrogen peroxide [17,27,28]. The use of probiotic supernatants without live cells might be more advantageous in preventing dental caries because, although live bacteria such as *Lactobacillus fermentum* NCINB 5221-inhibited *S. mutans* have attached to gingival epithelial cells as a co-aggregated complex with *S. mutans* [29]. Additionally, based on an investigation of the correlation between oral *lactobacilli* and dental caries, few *lactobacilli* were found in the oral cavities of caries-free children [30]. Thus, we examined the effect of SCSs from probiotic strains on *S. mutans*-induced preformed biofilm formation (Figure 2). *L. lactis* MG5125, *L. salivarius* MG4265, *Lactobacillus casei* MG311, and *Lactobacillus rhamnosus* MG316 supernatants showed a stronger reducing activity on the *S. mutans*-induced biofilm than other SCSs (*p* < 0.05).

This result suggests that *Lactobacillus* strains release bioactive substances that suppress *S. mutans*-induced biofilm formation, and this anti-biofilm formation effect is strain-specific. Dental plaque is a biofilm structure and consists of caries-related bacterial cells and an extracellular matrix [31]. Considering that the cariogenic activity of *S. mutans* is mainly due to its ability to adhere to teeth and produce exopolysaccharide [32], the biofilm-reducing activity of parabiotics is essential for the prevention of dental caries. Wasfi et al. [16] reported that the SCS of *L. salivarius* exerted the greatest reduction activity on the *S. mutans*-induced biofilm. The *L. salivarius*-mediated antibiofilm effect was attributed to significant downregulation of the *gtfB* gene of *S. mutans*. In this context, transcriptional *gtfs* gene regulation by the probiotic *Lactobacillus* strains positively contributed to the homeostasis of the oral microbiome [33].

Surface-active protein-rich biosurfactants derived from *lactobacilli* possibly decrease surface tension and inhibit biofilm formation [34,35].

*L. plantarum* lipoteichoic acid (a cell-wall component of gram-positive bacteria) suppressed *S. mutans*-induced biofilm formation by interfering with sucrose degradation, which is required for exopolysaccharide synthesis. The structural differences in the D-alanine repeating unit and glycolipid in lipoteichoic acid are responsible for selective inhibitory activity [36].

Based on the above results, the SCSs of *L. lactis* MG5125 and *L. salivarius* MG4265 have the potential to ameliorate dental caries by inhibiting the growth of *S. mutans* (Figure 1) and its biofilm formation (Figure 2).

### 2.3. Effect of SCSs on RANKL-Induced Osteoclast Differentiation

**Figure 3:**
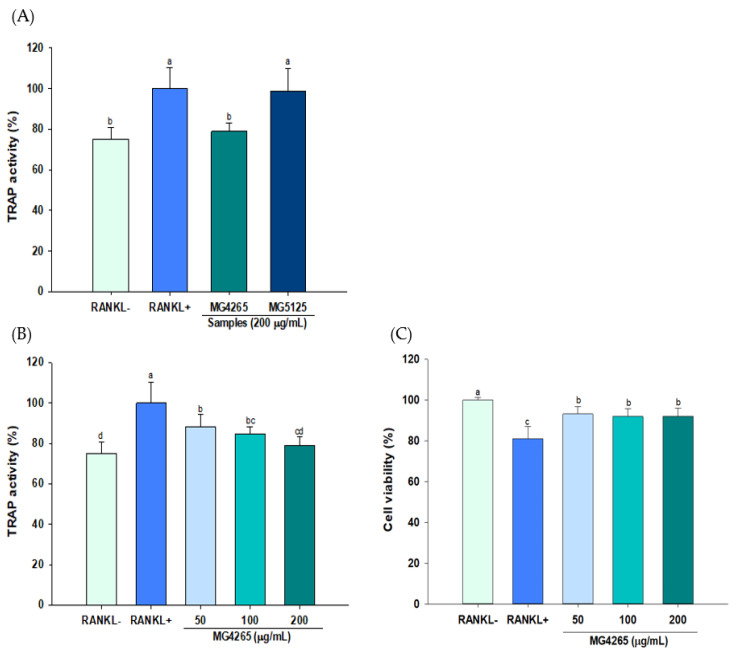
Effect of SCS produced from *L. salivarius* MG4265 and *L. lactis* MG5125 on (**A**) Tartrate-resistant acid phosphatase-positive (TRAP) activity, (**B**) concentration dependent effect, and (**C**) cell viability in nuclear factor κB ligand (RANKL) stimulated RAW 264.7 macrophages. Different letters (a–d) indicate significant difference at *p* < 0.05.

Osteoclasts are multinuclear cells that originate from precursor cells, such as monocyte or macrophage lineages, and play key roles in the bone loss from osteoporosis and periodontitis [37]. RAW 264.7 macrophages have been widely used as in vitro models of osteoclastogenesis since they are easily differentiated to osteoclasts by RANKL stimulation [38]. Tartrate-resistant acid phosphatase-positive (TRAP) activity was used to determine whether two preselected SCSs (*L. salivarius* MG4265 and *L. lactis* MG5125) also had a beneficial effect on RANKL-induced osteoclast formation in RAW 264.7 cells. *L. salivarius* MG4265 significantly reduced TRAP activity in RANKL-stimulated differentiated RAW 264.7 cells (*p* < 0.05, Figure 3A), whereas there was no significant difference in the case of *L. lactis* MG5125. The SCS of *L. salivarius* MG4265 significantly inhibited TRAP activity in a dose-dependent manner (*p* < 0.05, Figure 3B) and did not exhibit any cytotoxicity at the tested concentrations (*p* < 0.05, Figure 3C) These results indicate that the SCS of *L. salivarius* MG4265 actively suppressed RANKL-induced osteoclastogenesis. Thus, we further analyzed the effect of the SCS of *L. salivarius* MG4265 on osteoclast specific transcriptional factors and osteoclast associated genes expression.

### 2.4. Effect of the SCS of L. salivarius MG4265 on Osteoclast Specific Transcriptional Factors and Osteoclast-Associated Gene Expression

**Figure 4:**
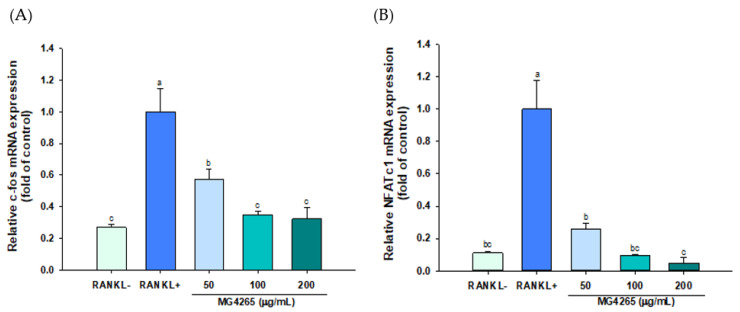
Effect of the SCS of *L. salivarius* MG4265 on osteoclast-specific transcription factors (**A**) *c-Fos* and (**B**) *NFATc1* mRNA expression in RANKL stimulated RAW 264.7 macrophages. Different letters (a–c) indicate significant difference at *p* < 0.05.

RANKL is an essential signaling molecule, and binding of RANKL to its receptor RANK induces the differentiation and activation of osteoclasts. This interaction stimulates the expression of osteoclast-specific key transcriptional effectors such as *c-Fos* and *NFATc1* [39]. The effect of the SCS of *L. salivarius* MG4265 on the gene expression of these two transcriptional factors was examined using qRT-PCR. The gene expression of *c-Fos* and nuclear factor of activated T cells cytoplasmic 1 (NFATc1) was decreased by 55% and 27%, respectively, in response to 50 μg/mL MG4265 treatment (Figure 4A,B).

*NFATc1* is a master regulator of osteoclast activation and modulates osteoclast adhesion and absorption of bone matrices through the upregulation of TRAP, cathepsin K, and matrix metalloproteinase-9 (MMP-9) [40,41]. The induction of NFATc1 is governed by *c-Fos* and *c-Fos* overexpression and has been recovered decreased NFATc1 expression by a p38-specific inhibitor [42,43].

**Figure 5:**
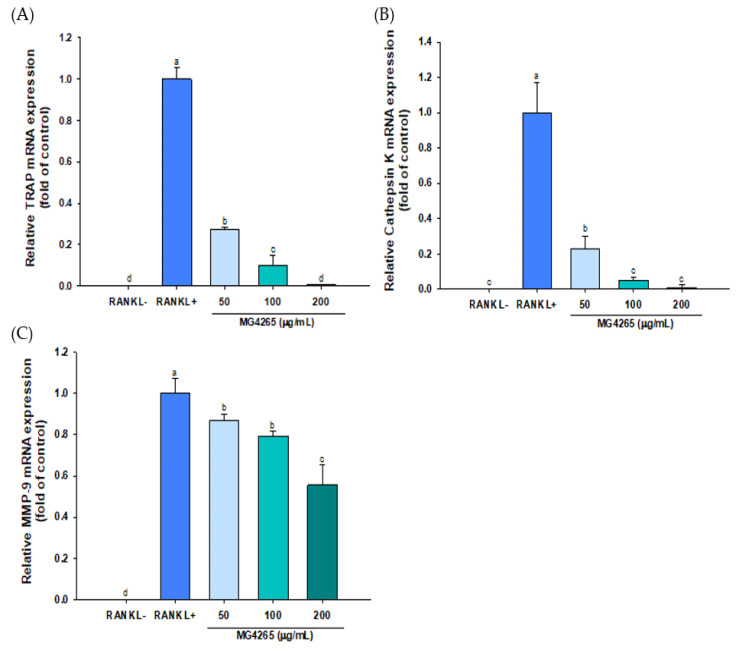
Effect of the SCS of *L. salivarius* MG4265 on osteoclast-specific (**A**) TRAP, (**B**) cathepsin K, and (**C**) MMP-9 mRNA expression in RANKL-stimulated RAW 264.7 macrophages. Different letters (a–d) indicate significant difference at *p* < 0.05.

Osteoclast-specific gene expression, including TRAP, cathepsin K, and *MMP-9* was also significantly decreased by treatment of MG4265 in a dose-dependent manner (Figure 5). Cathepsin K expression reflects the number of osteoclasts since it is mainly expressed in mature osteoclasts [44]. TRAP, cathepsin K, and *MMP-9* play a key role in osteoclast-mediated degradation of bone organic matrices such as collagen and osteopontin [45,46]. Downregulation of TRAP, cathepsin K, and *MMP-9* by *L. salivarius* MG4265 indicates that MG4265 can effectively inhibit bone resorption via regulation of osteoclast proteases. It has been demonstrated that the suppression of RANKL-mediated osteoclastogenesis in the RAW 264.7 cell model was highly correlated with decreased bone loss in ovariectomized rats [47].

### 2.5. Effect of the SCS of L. salivarius MG4265 on RANKL-Induced Mitogen Activated Protein Kinase (MAPKs) Activation

RANKL/RANK binding on osteoclast precursors such as RAW 264.7 cells recruits TNF receptor associated factor 6 (TRAF6), which mediates downstream signaling cascades. It is well known that MAPKs, such as c-Jun N-terminal kinase (JNKs), extracellular signal-regulated kinase (ERKs), and p38, are involved in osteoclast metabolism [48].

NF-κB is another crucial target of RANKL signaling, and selective inhibition of NF-κB nuclear translocation has been suggested as a therapeutic target for inflammatory bone loss [49]. Thus, the effect of the SCS of *L. salivarius* MG4265 on RANKL-mediated MAPK activation and NF-κB signaling were analyzed by Western blotting to investigate the molecular mechanisms related to c-Fos and NFATc1 downregulation.

**Figure 6:**
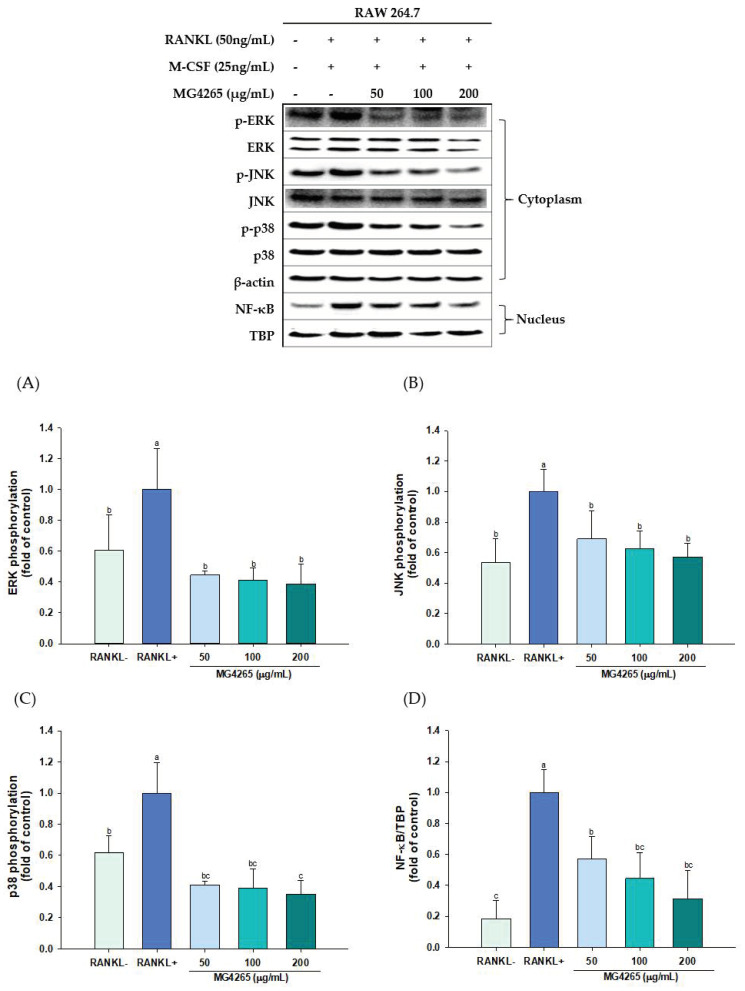
Effects of the SCS of *L. salivarius* MG4265 on (**A**) extracellular signal-regulated kinase (ERK), (**B**) c-Jun N-terminal kinase (JNK), (**C**) p38, and (**D**) nuclear NF-κB expression in RANKL-induced RAW 264.7 macrophages. Different letters (a–c) indicate significant difference at *p* < 0.05.

The phosphorylation of all MAPKs was significantly increased upon RANKL stimulation. Also, *L. salivarius* MG4265 treatment markedly inhibited the phosphorylation of all three MAPKs and the nuclear translocation of NF-κB (Figure 6). These results indicate that the SCS of *L. salivarius* MG4265 inhibited osteoclastogenesis by blocking the RANKL-activated MAPK and NF-κB signaling cascades, consequently downregulating *c-Fos* and *NFATc1* gene expression in osteoclast precursors.

The combination of RANKL and interleukin-1β, an inflammatory cytokine, synergistically activated ERK during osteoclastogenesis [50]. RANKL is a potent activator of JNK pathway, and the blocking of JNK signaling with a specific JNK inhibitor (SP600125) effectively reduced MMP expression and bone absorption in inflammatory arthritis rats [51]. Stimulation of p38 triggers the activation of osteoclastogenesis-specific transcriptional factors, such as NFATc1, and upregulates target gene expressions, such as cathepsin K [52].

NF-κB is normally confined to the cytoplasm in a complex with inhibitory κB (IκB), and it enters the nucleus upon degradation of IκB by RANKL or inflammatory cytokines. Liberated NF-κB binds to DNA target sites and expresses MMP-9, which leads to inflammatory bone damage [49,53].

There are only a few reports regarding parabiotics that attenuate periodontitis. In one study, the culture medium of *Lactobacillus reuteri* 6475 significantly suppressed RANKL-induced osteoclastogenesis in RAW 264.7 cells, and the authors suggested that lactobacillic acid was one of the active metabolites. GPR 120, a long-chain fatty acid receptor, was activated by the *L. reuteri* 6475 culture supernatant, and the MAPK pathway was involved in the suppression of osteoclastogenesis [54,55].

### 2.6. Effect of the SCS of L. salivarius MG4265 on HO-1 Induction

**Figure 7:**
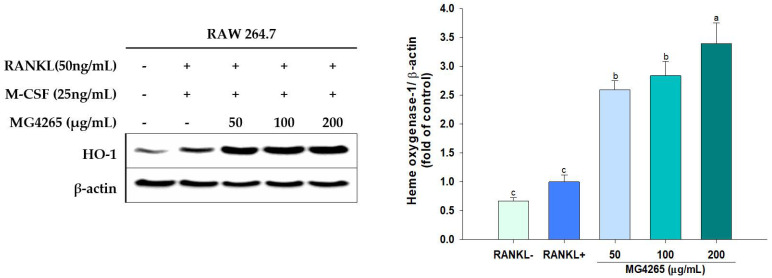
Effect of the SCS of *L. salivarius* MG4265 on HO-1 induction in RANKL-stimulated RAW 264.7 macrophages. Different letters (a–c) indicate significant difference at *p* < 0.05.

HO-1 is a stress-induced enzyme that modulates oxidative and inflammatory stress, and various phytochemicals inducing HO-1 have successfully inhibited RANKL-induced osteoclast differentiation [22,56]. RANKL exposure resulted in increased HO-1 expression, and it was further increased even with 50 μg/mL MG4265 treatment (Figure 7). The HO-1 induction was increased by 3.3-fold with treatment of 200 μg/mL of *L. salivarius* MG4265 (*p* < 0.05).

In a pathophysiological state, free heme content derived from myoglobin or other hemoproteins increases and iron atoms of hemes exert oxidative stress by catalyzing excess reactive oxygen species (ROS) production [57]. Periodontal disease is also under the influence of ROS produced from both the host and bacteria. Although the direct mechanisms are not clear, ROS is indirectly involved in periodontal tissue destruction by the stimulation of bone matrix proteinases [58]. In this regard, the SCS of *L. salivarius* MG4265 can alleviate periodontal disease by inhibiting *S. mutans* growth and its exopolysaccharide production. Mutan isolated from *S. mutans* directly induced differentiation of osteoclasts and accelerated alveolar bone loss in rats [59]. In addition, SCS of *L. salivarius* MG4265 have a positive effect on periodontal diseases through the modulation of osteoclastogenesis. *L. salivarius* MG4265 can decrease periodontal tissue damage by inhibiting the gene expression of encoding bone matrix degradation enzymes such as TRAP, cathepsin K, and MMP-9.

The efficacy of freeze-dried probiotic tablets containing *Lactobacillus salivarius* WB21 was demonstrated in randomized, double-blind, and placebo-controlled 8-week intervention study. Clinical parameters such as plaque index and probing pocket depth were significantly improved in the test group compared with those in the placebo group [60].

Lipoteichoic acid is a major virulence factor in *S. mutans.* It is involved in bacterial adhesion to dentin and eventually elicits host immune responses [61]. Considering that SCS of *L. salivarius* MG4265 actively decreased *S. mutans*-induced biofilm formation and RANKL-induced osteoclastogenesis, metabolites that were produced during fermentation possibly acted as effector molecules. The genus *Lactobacillus* secretes various metabolites, such as muropeptides, aggregation-promoting factors, bacteriocin, short-chain fatty acids, and trytophan-related metabolites [62,63], which may suppress biofilm formation and immune regulation.

SCFA acts as a key regulator of bone formation and absorption. Butyric acid and *L. rhamnosus* GG (LGG) exhibited equal activity in improving bone density [64]. The ethanol extract from *L. paracasei* subsp. *paracasei* NTU-fermented skim milk improved LPS-induced periodontal inflammation and decreased alveolar bone loss [21]. A mixture of tyrosine and lactic acid in a ratio of 3:1 was identified as an anti-periodontitis ingredient in the fermented extract [65].

Krzy’sciak et al. [66] reported that *Lactobacillus salivarius* HM6 Paradens significantly reduced the double-species biofilm of *S. mutans* and *Candida albicans* isolated from the dental caries of children. They further explained that *L. salivarius* might release agents that are able to inhibit cariogenic biofilm, but they could not identify active compounds that block biofilm formation or its mode of action. Characterization of the active compounds in SCS of *L. salivarius* MG4265 is required in future studies.

## 3. Materials and Methods

### 3.1. Strains and Cultivation

Eight probiotic strains (*Lactobacillus plantarum* MG207 (isolated from kimchi), *Lactobacillus paracasei* MG310 (isolated from fermented food), *L. casei* MG311 (isolated from fermented food), *L. rhamnosus* MG316 (isolated from infant feces), *L. salivarius* MG4265 (isolated from human origin), *L. lactis* MG5125 (isolated from fermented food), *L. fermentum* MG901 (human origin), and *Lactobacillus plantarum* MG989 (human origin)) were kindly provided from MEDIOGEN (Jecheon, Republic of Korea). *S. mutans* KCTC3065 was purchased from the Korean Collection for Type Culture (Daejeon, Republic of Korea). Probiotic strains were cultured in de Man, Rogosa, and Sharpe (MRS) broth (1% peptone, 0.8% meat extract, 0.4% yeast extract, 2% glucose, 0.5% sodium acetate, 0.2% dipotassium hydrogen phosphate, 0.02% magnesium sulfate heptahydrate, 0.005% manganese sulfate tetrahydrate, 0.02% triammonium citrate). *S. mutans* was cultured in brain-heart infusion (BHI) media (0.77% calf brains, 0.98% beef heart, 1% proteose peptone, 0.2% dextrose, 0.5% sodium chloride, 0.25% disodium phosphate). Probiotic strains were cultured at 37 °C in a CO_2_ incubator (Vision Scientific, Daejeon, Korea), and *S. mutans* was anaerobically cultured using GasPakTM EZ container systems (Becton Dickinson & Co., Sparks, MD, USA) at 37 °C.

### 3.2. Preparation of SCSs

The SCSs of probiotic strains were prepared according to the method of Lin et al. [17]. Probiotic strains were grown in MRS at 37 °C for 48 h. SCSs were obtained by centrifugation at 3470× *g* for 10 min followed by filtration using 0.2 µm filters (Advantec, Tokyo, Japan). The supernatants were lyophilized and used in the biofilm inhibition assay and cell culture study.

### 3.3. Effect of Probiotic Strains on the Growth of S. mutans

The effect of probiotic strains on the growth of *S. mutans* was examined using the method of Lin et al. with slight modifications [17]. The probiotic strains were grown in MRS at 37 °C for 48 h, and *S. mutans* was cultured in a BHI medium at 37 °C for 48 h under anaerobic stationary conditions. The concentrations of each probiotic strain and *S. mutans* were adjusted to 108 CFU/mL using phosphate-buffered saline (PBS). Finally, 50 µL of the *S. mutans* and each probiotic culture were combined and incubated under microaerophilic conditions at 37 °C for 24 h. After incubation, the suspensions were plated on MSB (Mitis Salivarius Sucrose Bacitracin) agar (Kisanbio, Seoul, Korea) and incubated at 37 °C for 24 h before counting the *S. mutans* colonies. The colony number of *S. mutans* in BHI medium without the probiotic strain was used as a negative control while PBS (50 µL) was used as blank control. The inhibition (%) was calculated as follows:

Inhibition (%) = (A − B) × 100/B

where A—colony number of the experimental group, B—negative control.

### 3.4. Effect of SCSs on the Growth of S. mutans

The effect of SCSs on the growth of *S. mutans* was measured by the method of Wasfi et al. [16]. *S. mutans* was grown at 37 °C in BHI broth and diluted to 10^8^ cells/mL with a BHI broth medium. SCSs (100 µL) from each probiotic strain were mixed with an equal volume of *S. mutans* suspension placed in a 96-well microplate. The plate was further incubated at 37 °C for 24 h and absorbance was measured at 600 nm using a microplate reader (Biotek Instruments Inc., Winooski, VT, USA). Sterilized MRS broth instead of SCS was used as a control.

### 3.5. Effect of SCSs on S. mutans-Induced Biofilm Formation

The culture of *S. mutans* was adjusted to 10^8^ CFU/mL in BHI containing 0.2% sucrose, and aliquots of the culture (100 µL) in the plate (96 well polystyrene plate; SPL Life Sciences Inc., Pocheon, Korea) were incubated overnight at 37 °C. The culture supernatant was replaced with the same volume of SCSs and incubated for another 48 h at 37 °C. After incubation, the medium was discarded, and the wells were thoroughly washed with sterilized distilled water. Then, the wells were stained with crystal violet solution (0.5%, 0.1 mL) for 10 min and washed. After drying, the absorbance of solubilized blue-colored biofilm in the wells was measured at 595 nm using the microplate reader [20].

### 3.6. RANKL-Induced Osteoclast Differentiation of Murine Osteoclast Progenitor RAW 264.7 Cells

The RAW 264.7 macrophages were purchased from American Type Culture Collection (ATCC, Manassas, VA, USA) and cultured and maintained as previously described [22]. RAW 264.7 cells were seeded (1 × 10^4^ cells/well) and incubated at 37 °C for 24 h. α-MEM (Welgene Inc., Daegu, Korea) containing RANKL (50 ng/mL; Prospec, Rehovot, Israel) and M-CSF (25 ng/mL, Sigma-Aldrich, St. Louis, MO, USA) was used as a medium for differentiation from RAW 264.7 macrophages into osteoclasts. The medium was changed every other day during the incubation period. Cell viability was evaluated using MTT [3-(4,5-dimethylthiazol-2-yl)-2,5-diphenyltetrazolium bromide] assay.

### 3.7. Tartrate-Resistant Acid Phosphatase-Positive (TRAP) Activity

RAW 264.7 cells were seeded (1 × 10^4^ cells/well) and incubated for another 8 days in the presence of the samples. After cells were lysed using a Triton X-100/saline solution, they were dispersed in citrate buffer containing 10 mM sodium tartrate and 10 mM *p*-nitrophenylphosphate (pH 4.7, 50 mM). TRAP activity was measured at 405 nm after incubation for 30 min at 37 °C.

### 3.8. RNA Extraction and Quantitative Real Time PCR (qRT-PCR)

RAW 264.7 cells were differentiated into osteoclasts for 42 h at various MG4265 SCS concentrations (50 μg/mL, 100 μg/mL, and 200 μg/mL). The differentiated cells were isolated using NucleoZOL (QIAGEN, Hilden, Germany) and RNA was extracted according to the manufacturer’s instruction. cDNA was synthesized using a cDNA kit (Applied Biosystems, Foster City, CA, USA), and quantitative relative expression of osteoclast-specific transcriptional factor genes (c-fos and NFATc1) and osteoclastogenesis-associated genes (TRAP, cathepsin K, and MMP-9) in RANKL-stimulated RAW 264.7 cells were analyzed by StepOne Plus RT-PCR system (Applied Biosystems, Foster City, CA, USA) using TaqMan^®^ Master mix (Applied Biosystems) [22]. The housekeeping gene β-actin was used as a reference gene to normalize the target-gene expressions.

### 3.9. Western Blotting Analysis

RAW 264.7 cells were seeded in a 6-well plate at 1 × 10^5^ cells/well and induced differentiation of osteoclast as described above. The cells were washed with cold PBS and lysed with a RIPA lysis buffer (ATTO, Tokyo, Japan) supplemented with phosphatase and protease inhibitors in a 98:1:1 (*v*/*v*/*v*) ratio. Nuclear proteins were isolated using NE-PER™ nuclear and cytoplasmic extraction reagents (Thermo Scientific, Rockford, IL, USA) according to the manufacturer’s instructions. Equal amounts of proteins, adjusted by the Bradford assay, were separated by SDS-PAGE (10% acrylamide) and then transferred onto polyvinylidene fluoride membranes (Millipore, Billerica, MA, USA). The immunoreactive proteins of interest were visualized and quantified by an enhanced chemiluminescence detection system (Bio-Rad, Hercules, CA, USA) following incubation with primary antibodies (c-Jun N-terminal kinase (JNK), extracellular signal-regulated kinase (ERK), p38, nuclear factor κB (NF-κB), heme oxygenase-1 (HO-1), β-actin, and TATA-binding protein (TBP); (Cell signaling Technology, Danvers, MA, USA)), followed by horse radish peroxidase conjugated secondary antibody (1:2500), as described previously [67]. The expressions of JNK, ERK, p38, and HO-1 were normalized to β-actin, whereas the expression of NF-κB in the nucleus was normalized to TBP.

### 3.10. Statistical Analysis

All quantitative experiments were conducted in triplicate. Data were expressed as the mean ± standard deviation and analyzed using SPSS 13.0 (SPSS, Inc., Chicago, IL, USA). One-way analysis of variance (ANOVA) and Duncan’s multiple comparison tests were used to determine significant differences (*p* < 0.05) among treatment means.

## 4. Conclusions

SCS of *L. salivarius* MG4265 strongly inhibited *S. mutans*-induced biofilm formation and RANKL-induced osteoclastogenesis. The suppression of RANKL-induced activation of the MAPKs and NF-κB pathways and upregulation of HO-1 expression inhibited the expression of transcriptional factors (c-fos and NFATc1) and osteoclastogenesis-related gene expression (TRAP, cathepsin K, and MMP-9). Therefore, SCS of *L. salivarius* MG4265 has great potential as a multifunctional oral health ingredient that inhibits not only biofilm formation but also suppresses the alveolar bone loss associated with periodontitis.

## Data Availability

Not applicable.

## Sample Availability

Samples of the compounds are available from the authors.
